# The clinicians’ view of food-related obstacles for treating eating disorders: A qualitative study

**DOI:** 10.29219/fnr.v67.8771

**Published:** 2023-01-25

**Authors:** Billy Langlet, Maria Nyberg, Karin Wendin, Modjtaba Zandian

**Affiliations:** 1 Division of Clinical Geriatrics, Center for Alzheimer Research, Department of Neurobiology, Care Sciences and Society, Karolinska Institutet, Stockholm, Sweden; 2 Food and Meals in Everyday Life (MEAL), Department of Food and Meal Science, Kristianstad University, Kristianstad, Sweden; 3 Department of Food Science, University of Copenhagen, Copenhagen, Denmark

**Keywords:** anorexia nervosa, bulimia nervosa, binge eating disorder, thematic analysis, focus groups, healthy, unhealthy, calories, taste, texture

## Abstract

**Background:**

Good health requires healthy eating. However, individuals with eating disorders, such as anorexia nervosa, require treatment to modify their dietary behaviours and prevent health complications. There is no consensus on the best treatment practices and treatment outcomes are usually poor. While normalising eating behaviour is a cornerstone in treatment, few studies have focused on eating and food-related obstacles to treatment.

**Objective:**

The aim of the study was to investigate clinicians’ perceived food-related obstacles to treatment of eating disorders (EDs).

**Design:**

Qualitative focus group discussions were conducted with clinicians involved in eating disorder treatment to get an understanding of their perceptions and beliefs regarding food and eating among eating disorder patients. Thematic analysis was used to find common patterns in the collected material.

**Results:**

From the thematic analysis the following five themes were identified: (1) ideas about healthy and unhealthy food, (2) calculating with calories, (3) taste, texture, and temperature as an excuse, (4) the problems with hidden ingredients and (5) the challenges of extra food.

**Discussion:**

All identified themes showed not only connections to each other but also some overlap. All themes were associated with a requirement of control, where food may be perceived as a threat, with the effects of food consumption resulting in a perceived net loss, rather than a gain. This mindset can greatly influence decision making.

**Conclusions:**

The results of this study are based on experience and practical knowledge that could improve future ED treatments by enhancing our understanding the challenges certain foods pose for patients. The results may also help to improve dietary plans by including and explaining challenges for patients at different stages of treatment. Future studies could further investigate the causes and best treatment practices for people suffering from EDs and other eating disturbances.

## Popular scientific summary

- As part of treating eating disorders, patients receive dietary recommendations. However, not many studies have focused on eating and food-related obstacles to treatment.
- Clinicians were interviewed in groups on the food-related obstacles eating disorder patients face during treatment.
- Five food-related obstacles (themes) were identified: (1) ideas about healthy and unhealthy food, (2) calculating with calories, (3) taste, texture, and temperature as an excuse, (4) the problems with hidden ingredients and (5) the challenges of extra food.

Eating and drinking seems simple but are complex processes. Preferences, choices, and behaviours related to food intake are determined by many factors and their interactions (1). An adequate energy intake and a balanced nutritious diet forms the foundation of a healthy life and helps protect against malnutrition, as well as non-communicable diseases (NCDs), such as diabetes, heart disease, stroke, and cancer (2). While most individuals consider it important to eat healthy, interpretations of recommendations and perceptions of what constitutes healthy foods varies greatly. A recent study showed that a healthy diet for some were to eat according to seasonal availability of food, for others it was the label information and for a third group it was sustainable consumption (3). Furthermore, emotions and social contexts, as well as self-determined motivation can influence food selection (4, 5). Disturbed eating behaviours can lead to eating disorders (EDs) as well as to other diseases (6–8).

ED are a collective term for diagnoses related to food, body perception and weight in which changed eating behaviour leads to decreased health (9). The three most recognised EDs are anorexia nervosa (AN), bulimia nervosa (BN) and binge-eating disorder (BED) (10). The aetiology is a complex interplay of sociocultural, biological, and psychological factors (11). EDs and the behaviours related to them can affect people of any gender, age, sexual orientation, and ethnicity and are most common in young women. A study conducted on 6,728 Americans between the ages of 9 and 14 found that 7.1% of boys and 14.4% of girls exhibited ED traits (12). Physiological effects from the most severe EDs, AN and BN are seen in almost all major body systems (13). Meanwhile, all EDs are associated with adverse psychological effects in the form of depression, mood and anxiety disorders and suicidal ideation, with an estimated 26% of patients having attempted suicide at some point (14).

There is currently no consensus on the best treatment practices for EDs (15) and most studies report poor treatment outcomes (16) and high relapse rates (17). For instance, one study examining the aetiology, assessment, and treatment of AN reported that only around 40% of the patients reached full recovery (17). The American Psychiatric Association’s Practice Guideline for the Treatment of Patients with EDs emphasise the importance of behavioural approaches for treating AN: ‘The goals of nutritional rehabilitation for seriously underweight patients are to restore weight, normalise eating patterns, achieve normal perceptions of hunger and satiety, and to correct biological and psychological sequelae of malnutrition’ (18). The dieticians role in nutrition intervention of EDs, as described by The American Dietetic Association, is to ensure diet quality and regular eating pattern, increased amount and variety of foods consumed, normalise perceptions of hunger and satiety, and provide a structured refeeding plan (16).

Several studies have explored clinicians’ attitudes and views on treating patients with EDs (19, 20). Most of these studies investigate the help-seeking process, expectations of care and appropriate referrals and collaboration versus opposition (21). Meanwhile, few studies investigate eating behaviour and food-related obstacles to treating EDs (10). What studies there are suggest ED patients avoid food in general (22–24), and high-caloric food in particular (25–27). Patients also tend to reduce food selection variety and fat intake, while overconsuming low-energy food, diet foods and drinks, water and coffee (28, 29). Due to a lack of information on eating behaviour, most dietetic treatment strategies focus on knowledge and awareness, such as informing patients to increase their energy intake and food variety (30). In a study by Biddiscombe et al., a behaviour-based approach was used, where ED patients were taken out to a café or restaurant to eat together in a group, assisted by an occupational therapist and a dietician, to model and support appropriate eating behaviour (31). This points at the importance of the therapists as well as clinicians and dieticians as role models in facilitating an improving eating behaviour among ED patients.

The aim of the study was to investigate clinicians’ perceived food-related obstacles to treatment of EDs.

## Material and methods

Qualitative focus groups were conducted with clinicians involved in ED treatment. Focus groups are often used when views, experiences, ideas, or perceptions on a certain topic are to be investigated (32). In a focus group, the views of the collective are essential as well as the dynamics of the group (33), and it is therefore considered a suitable method when the aim is to get an understanding of clinicians’ common perceptions and beliefs regarding food and eating among ED patients.

### Study context

Three clinics in Stockholm, Sweden were included in the study, with a total of nearly 30 fulltime clinicians, treating approximately 270 patients in 2021. Clinicians in this context are defined as working in inpatient- or outpatient care or as a dietician. All clinics employed the Mandometer method for treating EDs (34, 35).

The four pillars of the Mandometer method is retraining of normal eating and satiety responses (34), thermal treatment (36), restriction of physical activity (37) and social reconstruction to restore normal social interactions. Treatment starts with an eating evaluation where the patients baseline eating behaviour is measured. The baseline eating behaviour is used to create an individual treatment plan (35, 38). Depending on the severity of the ED patients are either admitted to inpatient care 24-h per day or to outpatient care.

The treatment plan includes a detailed meal plan, short- and long-term goals, expected weight gain, as well as a structured daily schedule for sleep, rest, and activities. The meal plan is created by a dietician and contains breakfast, lunch, dinner, and snack selections for patients. In the clinic, clinicians eat meals together with patients in a canteen area. The patient starts each meal by practicing putting the right amount of food on the plate, after which they consume the meal with feedback on eating rate (i.e. if they eat too fast or slow).

### Participants

The aim was to recruit between 16 and 20 clinicians involved in ED treatment between November 2020 and May 2021. Since the primary goal was to determine food-related behaviours that present an obstacle in treatment of EDs, inclusion criteria were working fulltime with ED patients as a clinician for >6-month. An email was sent out to clinicians who fulfilled the inclusion criteria. Clinicians who responded to the email met a researcher (BL) who explained the purpose of the study and collected a signed consent. After signing the consent form, participants provided researchers with information on their age and their years of experience working with EDs. Compensation for participation was given in the form of cinema tickets. In total, 16 participants were recruited, of which, 14 (88%) were female, the mean age was 36.5 (±8.4) years and the mean duration of working with EDs was 5.2 (±5.2) years.

### Focus groups

Participants were divided into four focus groups, trying to maintain an even distribution of participants working at the inpatient and outpatient ward and as dieticians for each group. When possible, co-workers were put in different groups. Each focus group took approximately 75 min and all were moderated by the same female researcher (MN). The researcher has a PhD, previous experience with focus groups and had no prior connection with the participants. The video conference software Zoom (Zoom Video Communications, California, USA) was used to facilitate each focus group and provide an audio capture of the discussion.

A focus group guide was used to assist the discussions taking place in the focus groups. The guide included questions about food that were perceived by the participants to be difficult to eat by ED patients, aided with images of various meals commonly served at ED clinics (Table 1, Supplementary file 1). The guide was divided into three parts: breakfast, lunch/dinner, and snacks. Specific questions asked for each part was: ‘What products are accepted during this meal type?’ and ‘are there any specific food qualities such as texture, taste, smell, visual appearance, or temperature that are more or less acceptable in certain patient groups?’. Of the many observable behavioural deviations of patients with EDs the questions in the guide focused on food-related behaviours, trying to operationalise them as the things that present an obstacle in treatment.

**Table 1:** Pictures of example meals shown to participants during the meeting

**Breakfast**
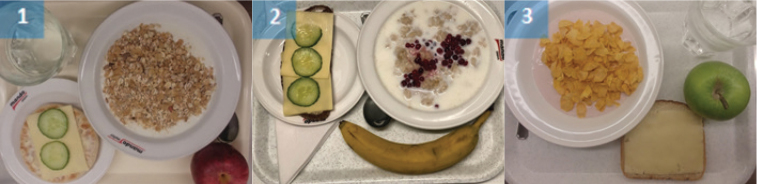
**Lunch and Dinner**
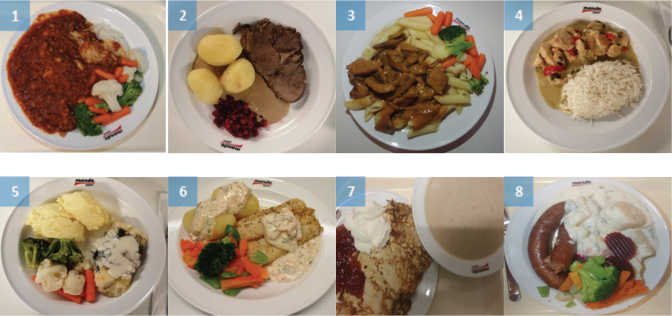
**Snack**
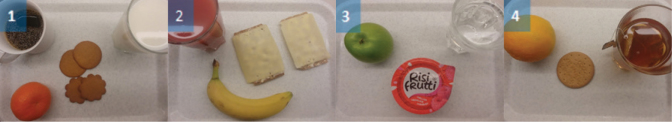
For a description of food components of each meal see Supplementary file 1.

### Thematic analysis

Conducting qualitative research is an ongoing, iterative process, and the analysis often starts already when collecting the empirical data and when the researcher listens to the narratives of the participants. The analysis was inspired by the thematical analysis presented by Braun and Clarke (39), focusing on finding central patterns and themes in the material. After conducting the focus groups the audio recordings were transcribed verbatim and read through several times searching for initial codes that related to the overall aim of the study. Codes were collated when they shared the same overall content and meaning. As the coding process continued these were refined and combined into potential themes, which were then reviewed and named to capture the essence and the overall content of the material.

Quotes in the results section were translated from Swedish to English by one author (BL). The abbreviation FG followed by a number signifies the focus group the quote belongs to.

### Ethics

This study did not handle sensitive personal information or law violations (3 §), nor did it include a physical intervention on a living or diseased person, include the study of biological material of a living or diseased person or intend to affect the participant physically or mentally s Therefore, the Swedish law for ethical review (2003:460 – Lagen om etikprövning av forskning som avser människor) was not applicable. Participation was voluntary, all participants signed consent forms before inclusion and were informed that they could, at any time, withdraw their consent.

## Results

The results highlight the participants perceived food-related obstacles to treatment of ED by focusing on the knowledge, practices, and ideas of the participants regarding food and eating behaviours of ED patients, and the patients’ preferences and acceptance of certain food. During the thematic analysis, five themes were identified: (1) ideas about healthy and unhealthy food, (2) calculating with calories, (3) taste, texture, and temperature as an excuse, 4) the problems with hidden ingredients and 5) the challenges of extra food.

### Ideas about healthy and unhealthy food

Participants agreed that the patient’s main reason for food and drink rejection is based on what is perceived to be ‘unhealthy’. The concept of unhealthy food is primarily food and drink with a high caloric content, but sometimes food is rejected based on perceived sweetness or fatness.

During the discussion of breakfast food, bread types was the most common topic. Participants agreed that crispbread is preferred over dark bread, which, in turn, is preferred over light bread:

Yes, I would say that they may rank [bread types]. Crispbread goes first, then dark bread is easier than light bread. Light bread comes last. (FG 4)

Suggested reasons for this selection order were that crispbread and dark bread was believed to have fewer calories, more fibres, and a greater satiety-inducing effect, while white bread was considered to be sweeter. The most common cold cuts used on bread are ham and cheese, due to perceptions of health regarding caloric content and fat. Porridge is a common selection, due to the perception of it being healthy (low calorie content) but can sometimes be rejected due to texture properties.

During lunch and dinner, the most preferred foods are vegetarian options, fish with boiled potatoes, or chicken fillet. Of fish types, white fish is preferred. Dishes based on fish are more often rejected, due to health claims, if they contain ‘skagenröra’ (mix of shrimps, mayonnaise, and dill) or are breaded. Many other lunch and dinner foods are considered unhealthy and result in rejection by patients, due to their high caloric content, such as sausage and stewed potatoes, pancakes with whipped cream and jam, and pasta dishes:

Yes. Many [patients] have the attitude that it [sausage] is unhealthy because it has a lower meat content and higher fat content than a piece of red meat. (FG 1)

The two food items most likely to cause rejection during snack times, due to health claims, are Risifrutti (rice dessert with vanilla flavour) and gingerbread cookies. Digestive biscuits are viewed as healthier, with some participants having observed that merely the name cookie (e.g. gingerbread cookie) could cause patients to reject them compared with biscuits that are viewed as healthier.

Of the fruits available in the clinic during breakfast and snack times, oranges and apples are the most popular. On occasion, oranges are rejected because they take too long to peel. Bananas are often rejected because they are considered unhealthy, based on energy content and sweetness. Green apples are selected more often than red, due to the sweeter red apples being associated with unhealthy food.

Water is often the beverage of choice. If water is not an option (due to the patients’ meal plan) fruit drinks (ProViva) are preferred over juice and lastly milk, motivated by ideas about health and low caloric content. For patients it is often more difficult to drink calories than to eat them, due to liquid calories being viewed as ‘unnecessary calories’.

### Calculating with calories

Patients are constantly counting calories in terms of what ingredients are included in a meal, how many food items are present on the plate, as well as the overall plate size. Participants agreed that patients most often base their meal selection on total amount of calories on the plate, rather than on specific food items present in the meal. An example of this thinking was illustrated by a participant when discussing the breakfast meals:

I would guess the caloric content is the same in all of these [meals] and a patient faced with this choice would sit and think about how many calories there are between these options. (FG 3)

For specific meals, low calorie soups, like tomato soup are selected over cream-based soups. When food is presented in units, like apples, patients are likely to take the smallest unit presented to reduce calory intake. Calory counting becomes more apparent with less complex meals, such as when patients choose between bread types. Patients may select a less preferred bread type, like white bread, if it has fewer cheese slices. Another strategy to reduce calorie intake is to select foods that can be dissected, like biscuits. The act of dissection is sometimes viewed as a ritualistic act of eating but participants describe it as a method for creating crumbles, which reduces calorie intake.

Another observation by participants is that the number of food products may influence meal selection. A meal with too many components sometimes causes rejection because it makes caloric calculations difficult. Another factor that can cause rejection is volume, where certain food items take up more space on the plate, which can result in a bias in calorie calculation and lead to rejection. Soup and rice were common examples of this phenomena:

(…) It can be very common for patients to experience difficulty with rice, for example, even though it is the same amount in grams, it may look like there is more on the plate (…). (FG 4)

Patients are quick to pick up new trends related to metabolism, with examples such as extensive use of tea and coffee, salt, or spices (such as pepper and cinnamon), which has caused the clinics to put limits on the amount of these products that are available to patients.

### Taste, texture, and temperature as an excuse

The participants argued that the patients often blame taste, texture, and temperature as a cause for rejecting certain foods, when the true criticism concerns caloric content. Foods that patients often complain about due to taste, texture, and temperature are meat, porridge, and banana. Some patients also complain about the temperature of foods, such as porridge, to avoid following the clinical protocol:

I believe that many of our patients may well use it as an excuse, for example, if the porridge is not hot, that then you will not be as inclined to complete the meal in the way we want. (FG 1)

The use of spices was not considered a problem and unrelated to the ED and the attitude towards spicy food was mainly thought to be due to personal preference. However, there were a few instances where participants thought taste, texture and temperature could affect the patients’ preference and acceptance of certain foods and drinks. Different textures can be difficult in the beginning of the treatment, especially if the patient has only been consuming nutritional drinks until recently. Risifrutti (see theme 1) is one of the food items patients struggle with, due to the creamy texture of the rice together with jam. Another food item that was perceived to be difficult for the patients to eat is meat, due to the visual presence of fat, but also due to the chewing requirements. Meanwhile, participants agreed that foods perceived as crispy, such as cornflakes, are easier to eat for patients than softer foods. The expected texture could also play a role, where soft cornflakes are more likely to be rejected by patients. Some patients alter food taste and texture, making the food less appetising by excessive use of salt and spices, or by mashing cornflakes in the yoghurt. Participants thought the reason for this was either because patients though food should not be enjoyed or that it is a strategy to avoid eating. In addition, participants thought that the times when texture was truly a problem for patients was when there were additional neuropsychiatric problems present.

### The problems with ‘hidden ingredients’

The participants stated that patients prefer foods where all ingredients are clearly visible and separated on the plate. When ingredients are not clearly visible patients often worry that there are ‘hidden ingredients’ of high caloric content in the food. An example is food fried in butter, such as meat, in which case the butter is the ‘hidden ingredient’. Even healthy foods suspected of containing ‘hidden ingredients’ could cause rejection, such as oven baked carrots, that if glossy could be mistaken for oily carrots. Other foods that are difficult for the patient are mashed or stewed potatoes, gratin with cheese, and lasagne. Sauces and stews are also difficult, because it is not apparent what the ingredients are, which often causes sauces that appear creamy to be rejected. Mixing multiple food items together also creates problems for patients, for similar reasons, because they cannot be sure of the volume of each food item. Covering food with sauce can also cause rejection because it may prevent patients from identifying all food components. Therefore, most patients prefer to get the sauce on the side of the food, which increases visibility and the feeling of control:

Many [patients] find it difficult when sauce, as in picture 1, when sauce or stew covers the rice. That you do not really see what everything is and how much everything is. And that it kind of blends together. (FG 3)

### The challenges of ‘extra food’

Another factor that often causes rejection of food by patients is if the food is considered ‘extra’, which in this context means food that is not part of the patient’s meal plan (i.e. obligatory part of the meal). This problem was most frequent with more severe ED cases in the inpatient ward. In the outpatient ward some patients instead use ‘extra’ vegetables to increase satiety. In the case of ‘extra food’, even foods that contain very few calories, like vegetables are often rejected:

Here [at the clinic] it is very rare that they choose vegetables just because it is outside their meal plan. Then it will be the case that you opt out of what is not required. (FG 3)

An example of this is that patients can avoid putting cucumber slices on bread, because it is not part of their meal plan. The reasoning still relates to calories and that the total amount of calories would be too much if cucumber was added to the meal.

## Discussion

This study aimed at identifying food-related obstacles to treatment of EDs, by employing focus groups with clinicians and finally conducting a thematic analysis of the qualitative data. The analysis generated five themes as obstacles to treatment, namely (1) ideas about healthy and unhealthy food, (2) calculating with calories, (3) taste, texture, and temperature as an excuse, 4) the problems with ‘hidden ingredients’ and (5) the challenges of ‘extra food’.

The finding that the concept of unhealthy food drives selection and rejection for ED patients, mainly in the form of high caloric food (25–27) and food high in fat content (29), is corroborated by subjective and objective studies. For example, in one study patients rated their urge to consume high calorie food significantly lower than healthy controls (26). Objective studies seldom use real food, but instead infer threat stimuli from attention tasks, such as the dot probe task, where ED patients seem to display attention vigilance for high-calorie food and attention avoidance for low-calorie food (40). Although previous studies use different food items for image-based tests, they rarely describe what food items are challenging for patients, but instead describe the quality of the food, for example, energy dense food (40). In this study, participants agreed that light bread, sausage and stewed potatoes, pancakes with whipped cream and jam, as well as pasta dishes were all difficult for ED patients to select and consume. Also, that using certain names for foods, such as cookie versus biscuit, can be enough to cause rejection by patients.

The discovery that patients calculate calories, although not always specifically stated (25–27), is in line with the observation that patients reject food of high caloric content. Something previous studies have not mentioned is that the calculation of calories, seems to be based on the entire meal and not specific food items of the meal. The practical effect being that some meals are selected over others, despite containing food items with high caloric content, if the total caloric content is lower than other available options. Another interesting observation by the participants was that failure to calculate calories for certain meals could result in rejection. For example, when a meal contains too many components or when it is difficult to estimate the volume of certain food items. Diet can influence mood and vice versa (41), which is why individuals without ED often use high caloric food, which may have an anxiolytic effect, for comfort (42). This is one of the reasons why calculating calories is a behaviour commonly used in dietary interventions for obesity (43). However, calculating calories may prevent recovery for certain ED patients and is something for clinicians to be watchful of. To our knowledge, this is the first time this food-related obstacle to ED treatment is described at such a detailed level.

It is well known that food texture has a large impact on food acceptance, especially among children (44). Studies have shown that textures have to be ‘right’ for different types of food and that familiarity, not only of the taste, but also of the texture, is of high importance (44). Results from this study suggest that ED patients mainly use taste, texture, and temperature as an excuse, to avoid certain foods. Furthermore, similar to the findings in this study, other studies have found that familiarity of texture for patients transitioning from liquid to solid food may be of particular interest (44). In addition, there are studies pointing out that the most aversive texture types are described as slimy or mushy (45), which is in line with the observation that ED patients may try to change texture attributes of certain foods into something inedible, for example, mashing corn flakes into yoghurt, which will make the texture ‘slimy’. Inedibility may also be induced by addition of high amounts of spices, for example, spices impacting the trigeminal nerve such as chili pepper or ginger, causing a painful mouth burning sensation (46). It is important to increase the understanding of patients’ strategies of avoidance, to ensure that patients are trusted in situations where taste, texture, and temperature are problems, but that clinicians can identify situations where avoidance strategies are a risk to the patients’ recovery (47). Also, with EDs such as avoidant restrictive feeding and ED and AN it is important to understand the underlying reason for food avoidance.

Visual appearance of a food or a dish is of great importance for the first evaluation of the food. It creates expectations of the food experience (e.g. taste and texture) (48). Furthermore, Rowley and Spence (49) showed that the way in which food is presented visually on the plate has a substantial influence on consumer’s perception. In this study, clinicians reported that patients preferred clearly visible and separated food items on the plate. Sauces, mixes of food and dishes where unwanted ingredients may hide were often rejected. It was mentioned that an explanation could be diminished control of what is on the plate. This is in parallel with elderly people who prefer food clearly visible on the plate to be in control of what to eat or not (50). There are several studies that describe an avoidance of certain food items (sometimes referred to as fear foods) in EDs, resulting in a reduction of diet diversity (51). However, only a few studies mention the behaviour of clearly separating food items on the plate and we found no studies which described the problem of hidden ingredients for ED patients.

The challenge of extra food, especially for more severe ED cases, is likely caused by a fear of gaining weight. However, it could also be influenced by cognitive inflexibility, which is common in ED patients (52), where patients have a problem to adapt to what is perceived as changes in the meal plan. Therefore, extra care should be taken when preparing meal plans for an ED patient, because small changes to the plan may cause substantial problems to treatment.

All the identified themes showed not only connections to each other but also some overlaps, for example, the difficulty of calculating calories when ingredients are hidden. However, clinicians discussed them as separate food-related obstacles, which is why they make out the five themes in this study. All themes were associated with a requirement of control, likely stemming from food being perceived as threats and something to avoid by the ED patients (47). For example, when food is hidden or for complex meal compositions where calorie calculation is difficult. As a result, each theme reduces the diet diversity for the ED patient and narrows selection. Different models and theories of food choice are used to understand how healthy individuals actively choose food in everyday life (1). However, in understanding eating behaviour among people with EDs this study has demonstrated the need to understand how food choice is influenced by rejection. One reason for food rejection, rather than selection, for ED patients could be that they view food consumption and its effects as a loss, rather than a gain. This mindset can greatly influence decision making as described in a monetary setting by McGraw et al. (53).

A strength of the study was that all clinicians had been working for more than 6 months with EDs and most had worked for several years treating EDs. It is also important to note that the results represent clinicians’ perceptions of food-related obstacles for ED patients. And while it is important to understand a clinicians’ view on EDs, it may not accurately reflect the perception of ED patients or the ‘true’ problem.

Since eating behaviour normalisation is part of ED treatment, understanding the difficulties certain foods present to ED patients is important in interactions with ED patients. It also enables dieticians to create dietary plans with adequate challenges for ED patients at different stages of treatment. In addition, the findings of this study can be used to design experimental studies to objectively evaluate the effect certain themes, such as calorie calculation, have on selection and rejection for ED patients. Future studies could also investigate the causes and best treatment practices for the food-related obstacles.

## Supplementary Material

The clinicians’ view of food-related obstacles for treating eating disorders: A qualitative study
