# Supplementary material for: The clinicians’ view of food-related obstacles for treating eating disorders: A qualitative study
Source: Food Nutr Res. 2023 Jan 25;67:10.29219/fnr.v67.8771. doi: 10.29219/fnr.v67.8771 (PMC9899047; doi:10.29219/fnr.v67.8771)
Supplement: The clinicians’ view of food-related obstacles for treating eating disorders: A qualitative study [file FNR-67-8771-s001.docx]

Meal types

Table 1. Description of example meals shown to participants during the meeting.

| Meal | Food items |
| --- | --- |
| Breakfast |  |
| Meal 1 | A bowl of processed sour milk with muesli, 1 white bread with 1 cheese slice and 2 cucumber slices, 1 red apple, a glass of water |
| Meal 2 | Oatmeal with milk, dark bread with 2 cheese slices and 3 cucumber slices, 1 banana |
| Meal 3 | Yoghurt with cornflakes, white bread with 2 cheese slices, 1 green apple, a glass of water |
| Lunch/Dinner |  |
| Meal 1 | Nasi Goreng (rice, vegetables, and mincemeat) |
| Meal 2 | Beef with boiled potatoes, cream sauce, and lingonberries |
| Meal 3 | Beef stew with pasta and vegetables |
| Meal 4 | Chicken with rice, vegetables, and green curry sauce |
| Meal 5 | Haddock with potato mash, vegetables, and dill sauce |
| Meal 6 | Cod with boiled potatoes, shrimp sauce and vegetables |
| Meal 7 | Plate of pancakes with whipped cream and jam, bowl of pea soup |
| Meal 8 | Sausages with potato gratin, beetroots, and other vegetables |
| Snack |  |
| Meal 1 | 4 gingerbread cookies, 1 clementine, 1 cup of coffee, 1 glass of milk |
| Meal 2 | 2 crispbreads each with 1 cheese slice, 1 banana, 1 glass of fruit drink |
| Meal 3 | 1 Risifrutti, 1 green apple, 1 glass of water |
| Meal 4 | 1 Digestive biscuit, 1 orange, 1 cup of tea |
